# Incentives, Nudges and the Burden of Proof in Ethical Argument

**DOI:** 10.1136/medethics-2017-104198

**Published:** 2017-02-22

**Authors:** Richard E Ashcroft

**Keywords:** Ethics

Behaviour change is increasingly prominent in public health and social policy worldwide. The papers contributed to this special issue cite numerous examples. The kinds of intervention highlighted in this special issue range from conditional cash transfers, which make use of traditional models of social welfare payments modified to encourage particular behaviours such as school attendance, to incentives to quit smoking or complete vaccination schedules, to “nudges” which seek to affect decision-making by semi-conscious or unconscious “altering defaults” in the framing of choices. Sometimes the intention behind these interventions is to encourage agents to do things which they know they want to do, or ought to do, but find difficult to do in practice; sometimes the intention is actually to alter agents' preferences. Sometimes the intended beneficiary of the change in behaviour is the agent him or herself; sometimes it is a third party known to the agent (such as a child of the agent); sometimes it is for the benefit of more distant third parties; and sometimes it is for the general, common, or public good, however conceived. Here I am drawing attention to the sheer diversity of aims and intentions and targets of interventions. It is not, in my view, useful to make broad brush assumptions about “behaviour change” interventions as if they were homogeneous in type, design, intended effect, mechanism of action, or underlying ethical norms.

There has been considerable ethical debate about the ethics of behaviour change interventions, not only because these interventions are newly fashionable, but also because they also because they seem to set a number of ethical alarm bells ringing. If one is mainly concerned with autonomy and liberty, then they seem to involve methods of suborning the will of the individual agent. If one is concerned primarily with justice, they seem to involve unfair burdens on the economically, socially or psychologically vulnerable. If one is concerned with the classical values of public health as collective action at the societal level, they seem to involve socially atomistic and individualistic assumptions which corrode solidarity. And so on.

And yet none of this is easy or straightforward of proof. For example: incentive schemes are explicitly addressed to agents, whereas nudges are not. Yet nudges can be reflectively endorsed by agents, if they are drawn to their attention, while incentive schemes may partly depend on unconscious signals and expectations. So while at first sight incentives are more autonomy-respecting and nudges are more paternalistic, when we focus on the empirical psychology involved in their mechanisms of action, things are much less clear cut. Similarly, we have normative paradoxes such as this: on the one hand, incentives to quit smoking will, it is argued by many critics, unfairly target the poor, who need the money more, whose marginal utility of money is higher, and who are more likely to be patronised and disapproved of by the “liberal elite”. On the other, the poor are more likely to smoke, have a higher burden of disease in general, and are typically less able to access healthcare; so a concern with health inequality should reinforce a commitment to an intervention which is more likely to benefit the worst off. And while this paradox in what justice requires needs considerable theoretical work to resolve, we then further have the problems that we have weak evidence that such interventions work even in controlled trials, and practically speaking no evidence that any of these effects are detectable in real life, or that, if detectable, they are persistent.

In effect, we have an ethical debate whose central feature is a set of arguments about the rightness or wrongness of interventions, on the assumption that they have significant, measurable and occurrent effects, but in the absence of much reliable evidence either that they do have such effects, or that they have those effects following the mechanisms of action we assume them to have. It is reasonable and important to have such debates in advance of the experimental evidence – this is a (somewhat neglected) branch of research ethics. It is less clear that it is reasonable to do so when we do have evidence which suggests that our working premises are in fact unsupported by that evidence.

However, a different sort of ethical debate remains highly pertinent and worthwhile, and the papers in this special issue do make valuable contributions to it. Even though the experimental evidence for many behaviour change interventions is negative or at best unimpressive (there are some exceptions, I concede, particularly in treatment of drug addiction), policymakers, commercial companies and many practitioners in public health and social policy continue to believe otherwise. Several important questions arise: why do they do so? Why are the ethical assumptions made about such interventions so impervious to new information? What other normative and policy objectives are served by promoting these interventions, and these ethical frameworks, beyond the explicit goals of behaviour change (which are largely not met)? And, more generally, what are the defaults of burden of proof in such debates, and how, if at all, are they rational and justifiable?

